# Tincture of Chloride of Iron

**Published:** 1881-09

**Authors:** 


### TINCTURE OF CLORIDE OF IRON.

Dr. H. Hagar recommends that Tincture Terri Chloridi be mixed with simple syrup and then with milk. This mixture does not affect the teeth, nor is the usual styptic taste apparent.—*Druggist's Circular.*

